# Catalysis by Metal-Oxide Nanostructures

**DOI:** 10.3390/nano14050415

**Published:** 2024-02-24

**Authors:** Sónia Alexandra Correia Carabineiro

**Affiliations:** LAQV-REQUIMTE, Department of Chemistry, NOVA School of Science and Technology, Universidade NOVA de Lisboa, 2829-516 Caparica, Portugal; sonia.carabineiro@fct.unl.pt

Catalysis is an important field dealing with innovation, sustainability, and materials science that has been witnessing remarkable advancements through nanotechnology [1,2,3,4,5,6,7,8,9,10]. This Special Issue of *Nanomaterials* deals with research on “Catalysis by Metal-Oxide Nanostructures”, featuring several papers that illustrate the diverse and impactful applications of these nanostructures in catalytic processes.

In the pursuit of selective hydrogenation, Ni-modified Ag/SiO_2_ catalysts have emerged as promising candidates for converting dimethyl oxalate to methyl glycolate (contribution 1). This work not only shows the catalytic efficiency of metal-modified nanostructures but also emphasizes the importance of selectivity in sustainable chemical transformations.

Electrocatalysis is shown through the exploration of hollow CoP/FeP_4_ heterostructural nanorods interwoven by carbon nanotubes (CNTs) as highly efficient electrocatalysts for oxygen evolution reactions (contribution 2). This study underscores the synergistic effects of heterostructures and CNTs in enhancing electrocatalytic performance, paving the way for advancements in energy conversion technologies.

A magnetic core–shell iron(II) C-scorpionate catalyst, exhibiting catalytic efficiency under unconventional oxidation conditions, adds a unique dimension to our understanding of the versatility of metal-oxide nanostructures in challenging reaction environments (contribution 3).

Functionalizing magnetic nanoparticles with bioactive compounds is also explored through the development of chitosan-functionalized magnetic nanoparticles (contribution 4). This work not only presents a novel approach to nanocatalyst design but also opens avenues for combining catalysis with therapeutic or biocompatible functionalities.

The synergy between carbon quantum dots and (002)-oriented Bi_2_O_2_CO_3_ composites is harnessed for enhanced photocatalytic removal of toluene in air (contribution 5). This research showcases the potential of tailored nanostructures in addressing environmental challenges through advanced photocatalysis.

The impact of thermal treatment on Nb_2_O_5_ in glucose dehydration to 5-hydroxymethylfurfural in water is investigated, shedding light on the thermal stability and catalytic activity of metal oxides in biomass conversion (contribution 6).

A one-step synthesis of tetragonal-CuBi_2_O_4_/amorphous-BiFeO_3_ heterojunctions demonstrates improved charge separation and enhanced photocatalytic properties, offering insights into designing efficient nanostructured heterojunctions for catalytic applications (contribution 7).

Cationic magnetite nanoparticles emerge as agents for increasing siRNA hybridization rates, adding a nanocatalytic perspective to nucleic acid interactions (contribution 8).

Turning waste into a resource, biochars and activated carbons from olive oil industry residues are explored as supports for Fe-catalysts in the heterogeneous Fenton-like treatment of simulated olive mill wastewater, showcasing the potential of sustainable catalyst supports (contribution 9).

Investigations on Mn_3_O_4_-coated Ru nanoparticles for partial hydrogenation of benzene towards cyclohexene production reveal the interesting interplay between metal oxides and additives in catalytic processes (contribution 10).

CO_2_ hydrogenation over nanoceria-supported transition-metal catalysts unveils the role of ceria’s morphology and active phase nature, providing critical insights for designing efficient catalysts for CO_2_ conversion (contribution 11).

Pd/UiO-66-v catalysts were fabricated for the conversion of furfuryl alcohol to tetrahydrofurfuryl alcohol under mild conditions in water, showing the potential of metal–organic-framework-supported nanocatalysts (contribution 12).

A bimetal CuFe_2_O_4_ oxide redox-active nanocatalyst is synthesized for the oxidation of pinene to renewable aroma oxygenates, bridging the gap between redox-active materials and catalytic transformations (contribution 13).

Copper ferrite nanosphere composites are explored as Fenton catalysts for the removal of phenolic compounds from water, highlighting the potential of metal-oxide nanocatalysts in water treatment applications (contribution 14).

Molybdenum disulfide (MoS_2_) synthesized through a common hydrothermal method exhibits coexisting 1T and 2H phases for efficient hydrogen evolution reactions, contributing to the understanding of phase-dependent catalytic activity (contribution 15).

The electronic state of gold is investigated for its effect on the catalytic performance of nano gold catalysts in n-octanol oxidation (contribution 16). This study provides crucial insights into tailoring gold catalysts for specific oxidation reactions.

Supported gold nanoparticles exhibit catalytic activity in the peroxidative and aerobic oxidation of 1-phenylethanol under mild conditions, emphasizing the versatility of gold-based nanostructures in green oxidation methodologies (contribution 17).

The cooperative effects between metal and support in Au/VPO are investigated for the aerobic oxidation of benzyl alcohol to benzyl benzoate, showcasing the significance of metal–support interactions in catalytic processes (contribution 18).

This Special Issue also includes a review on catalytic methane decomposition to carbon nanostructures and CO_x_-free hydrogen, offering a comprehensive overview of this intriguing field (contribution 19).

As the Guest Editor of this Special Issue, I thank all the authors for their exceptional contributions and the dedicated MDPI staff members for the important editorial support. As we dive deeper into the catalytic potential of metal-oxide nanostructures, these research papers show us new frontiers in sustainable and efficient catalysis. 

## List of Contributions

1. Cheng, S.; Meng, T.; Mao, D.; Guo, X.; Yu, J.; Ma, Z. Ni-Modified Ag/SiO_2_ Catalysts for Selective Hydrogenation of Dimethyl Oxalate to Methyl Glycolate. *Nanomaterials* **2022**, *12*, 407.
2. Liu, Y.; Li, Y.; Wu, Q.; Su, Z.; Wang, B.; Chen, Y.; Wang, S. Hollow CoP/FeP_4_ Heterostructural Nanorods Interwoven by CNT as a Highly Efficient Electrocatalyst for Oxygen Evolution Reactions. *Nanomaterials* **2021**, *11*, 1450.
3. Matias, I.A.S.; Ribeiro, A.P.C.; Ferraria, A.M.; Rego, A.M.B.d.; Martins, L.M.D.R.S. Catalytic Performance of a Magnetic Core-Shell Iron(II) C-Scorpionate under Unconventional Oxidation Conditions. *Nanomaterials* **2020**, *10*, 2111.
4. Hojnik Podrepšek, G.; Knez, Ž.; Leitgeb, M. Development of Chitosan Functionalized Magnetic Nanoparticles with Bioactive Compounds. *Nanomaterials* **2020**, *10*, 1913.
5. Ding, J.; Wang, H.; Luo, Y.; Xu, Y.; Liu, J.; Lin, R.; Gao, Y.; Lin, Y. Carbon Quantum Dots Modified (002) Oriented Bi_2_O_2_CO_3_ Composites with Enhanced Photocatalytic Removal of Toluene in Air. *Nanomaterials* **2020**, *10*, 1795.
6. Morawa Eblagon, K.; Malaika, A.; Ptaszynska, K.; Pereira, M.F.R.; Figueiredo, J.L. Impact of Thermal Treatment of Nb_2_O_5_ on Its Performance in Glucose Dehydration to 5-Hydroxymethylfurfural in Water. *Nanomaterials* **2020**, *10*, 1685.
7. Cai, F.; Zhang, T.; Liu, Q.; Guo, P.; Lei, Y.; Wang, Y.; Wang, F. One Step Synthesis of Tetragonal-CuBi_2_O_4_/Amorphous-BiFeO_3_ Heterojunction with Improved Charge Separation and Enhanced Photocatalytic Properties. *Nanomaterials* **2020**, *10*, 1514.
8. Prilepskii, A.Y.; Kalnin, A.Y.; Fakhardo, A.F.; Anastasova, E.I.; Nedorezova, D.D.; Antonov, G.A.; Vinogradov, V.V. Cationic Magnetite Nanoparticles for Increasing siRNA Hybridization Rates. *Nanomaterials* **2020**, *10*, 1018.
9. Esteves, B.M.; Morales-Torres, S.; Maldonado-Hódar, F.J.; Madeira, L.M. Fitting Biochars and Activated Carbons from Residues of the Olive Oil Industry as Supports of Fe-Catalysts for the Heterogeneous Fenton-Like Treatment of Simulated Olive Mill Wastewater. *Nanomaterials* **2020**, *10*, 876.
10. Liu, X.; Chen, Z.; Sun, H.; Chen, L.; Peng, Z.; Liu, Z. Investigation on Mn_3_O_4_ Coated Ru Nanoparticles for Partial Hydrogenation of Benzene towards Cyclohexene Production Using ZnSO_4_, MnSO_4_ and FeSO_4_ as Reaction Additives. *Nanomaterials* **2020**, *10*, 809.
11. Konsolakis, M.; Lykaki, M.; Stefa, S.; Carabineiro, S.A.C.; Varvoutis, G.; Papista, E.; Marnellos, G.E. CO_2_ Hydrogenation over Nanoceria-Supported Transition Metal Catalysts: Role of Ceria Morphology (Nanorods versus Nanocubes) and Active Phase Nature (Co versus Cu). *Nanomaterials* **2019**, *9*, 1739.
12. Yang, Y.; Deng, D.; Sui, D.; Xie, Y.; Li, D.; Duan, Y. Facile Preparation of Pd/UiO-66-v for the Conversion of Furfuryl Alcohol to Tetrahydrofurfuryl Alcohol under Mild Conditions in Water. *Nanomaterials* **2019**, *9*, 1698.
13. Mdletshe, L.S.; Makgwane, P.R.; Ray, S.S. Fabrication of Bimetal CuFe_2_O_4_ Oxide Redox-Active Nanocatalyst for Oxidation of Pinene to Renewable Aroma Oxygenates. *Nanomaterials* **2019**, *9*, 1140.
14. Moreno-Castilla, C.; López-Ramón, M.V.; Fontecha-Cámara, M.Á.; Álvarez, M.A.; Mateus, L. Removal of Phenolic Compounds from Water Using Copper Ferrite Nanosphere Composites as Fenton Catalysts. *Nanomaterials* **2019**, *9*, 901.
15. Yao, Y.; Ao, K.; Lv, P.; Wei, Q. MoS_2_ Coexisting in 1T and 2H Phases Synthesized by Common Hydrothermal Method for Hydrogen Evolution Reaction. *Nanomaterials* **2019**, *9*, 844.
16. Pakrieva, E.; Kolobova, E.; Kotolevich, Y.; Pascual, L.; Carabineiro, S.A.C.; Kharlanov, A.N.; Pichugina, D.; Nikitina, N.; German, D.; Zepeda Partida, T.A.; et al. Effect of Gold Electronic State on the Catalytic Performance of Nano Gold Catalysts in n-Octanol Oxidation. *Nanomaterials* **2020**, *10*, 880.
17. Pakrieva, E.; P. C. Ribeiro, A.; Kolobova, E.; M. D. R. S. Martins, L.; A. C. Carabineiro, S.; German, D.; Pichugina, D.; Jiang, C.; J. L. Pombeiro, A.; Bogdanchikova, N.; et al. Supported Gold Nanoparticles as Catalysts in Peroxidative and Aerobic Oxidation of 1-Phenylethanol under Mild Conditions. *Nanomaterials* **2020**, *10*, 151.
18. Campisi, S.; Ferri, M.; Chan-Thaw, C.E.; Sanchez Trujillo, F.J.; Motta, D.; Tabanelli, T.; Dimitratos, N.; Villa, A. Metal-Support Cooperative Effects in Au/VPO for the Aerobic Oxidation of Benzyl Alcohol to Benzyl Benzoate. *Nanomaterials* **2019**, *9*, 299.
19. Gamal, A.; Eid, K.; El-Naas, M.H.; Kumar, D.; Kumar, A. Catalytic Methane Decomposition to Carbon Nanostructures and COx-Free Hydrogen: A Mini-Review. *Nanomaterials* **2021**, *11*, 1226.

## References

[1] Anastas P.T., Bartlett L.B., Kirchhoff M.M., Williamson T.C. (2000). The role of catalysis in the design, development, and implementation of green chemistry. Catal. Today.

[2] Schmidt F., Baerns M. (2004). The Importance of Catalysis in the Chemical and Non-Chemical Industries. Basic Principles in Applied Catalysis.

[3] Busacca C.A., Fandrick D.R., Song J.J., Senanayake C.H. (2011). The Growing Impact of Catalysis in the Pharmaceutical Industry. Adv. Synth. Catal..

[4] Thomas J.M. (2012). The societal significance of catalysis and the growing practical importance of single-site heterogeneous catalysts. Proc. R. Soc. A Math. Phys. Eng. Sci..

[5] Ball P. (2015). Catalysis: Facing the future—An interview with Gerhard Ertl and Avelino Corma. Natl. Sci. Rev..

[6] Munnik P., de Jongh P.E., de Jong K.P. (2015). Recent Developments in the Synthesis of Supported Catalysts. Chem. Rev..

[7] Catlow C.R., Davidson M., Hardacre C., Hutchings G.J. (2016). Catalysis making the world a better place. Philos. Trans. R. Soc. Ser. A Math. Phys. Eng. Sci..

[8] Ranade V.V., Joshi S.S. (2016). Chapter 1—Catalysis and Catalytic Processes. Industrial Catalytic Processes for Fine and Specialty Chemicals.

[9] Thomas J.M. (2018). The enduring relevance and academic fascination of catalysis. Nat. Catal..

[10] Astruc D. (2020). Introduction: Nanoparticles in Catalysis. Chem. Rev..

